# Challenges and opportunities for improved contact tracing in Ghana: experiences from Coronavirus disease-2019-related contact tracing in the Bono region

**DOI:** 10.1186/s12879-023-08317-6

**Published:** 2023-05-18

**Authors:** Isaac Tachie Asare, Mbuyiselo Douglas, Gideon Kye-Duodu, Emmanuel Manu

**Affiliations:** 1 Department of Population and Behavioural Sciences, Fred N. Binka School of Public Health, University of Health and Allied Sciences, Hohoe, Ghana; 2 Department of Public Health, Faculty of Health Sciences, Walter Sisulu University, Private Bag X1, Mthatha, 5117 South Africa; 3 Department of Epidemiology and Biostatistics, Fred N. Binka School of Public Health, University of Health and Allied Sciences, Hohoe, Ghana

**Keywords:** Contact tracing, Bono region, COVID-19 prevention, Emergency preparedness, Pandemic control

## Abstract

**Background:**

In Ghana, contact tracing received heightened attention in the fight against the COVID-19 pandemic during its peak period. Despite the successes achieved, numerous challenges continue to limit the efforts of contact tracing in completely curtailing the effect of the pandemic. Despite these challenges, there are still opportunities that could be harnessed from the COVID-19 contact tracing experience for future eventualities. This study thus identified the challenges and opportunities associated with COVID-19 contact tracing in the Bono Region of Ghana.

**Methods:**

Using a focus group discussion (FGD) approach, an exploratory qualitative design was conducted in six selected districts of the Bono region of Ghana in this study. The purposeful sampling technique was employed to recruit 39 contact tracers who were grouped into six focus groups. A thematic content analysis approach via ATLAS ti version 9.0 software was used to analyse the data and presented under two broad themes.

**Results:**

The discussants reported twelve (12) challenges that hindered effective contact tracing in the Bono region. These include inadequate personal protective equipment, harassment by contacts, politicisation of the discourse around the disease, stigmatization, delays in processing test results, poor remuneration and lack of insurance package, inadequate staffing, difficulty in locating contacts, poor quarantine practices, poor education on COVID-19, language barrier and transportation challenges. Opportunities for improving contact tracing include cooperation, awareness creation, leveraging on knowledge gained in contact tracing, and effective emergency plans for future pandemics.

**Conclusion:**

There is a need for health authorities, particularly in the region, and the state as a whole to address contact tracing-related challenges while simultaneously harnessing the recommended opportunities for improved contact tracing in the future for effective pandemic control.

**Supplementary Information:**

The online version contains supplementary material available at 10.1186/s12879-023-08317-6.

## Introduction

The outbreak of the coronavirus disease (COVID-19) in 2019 necessitated the strategic implementation of diverse measures around the world to curb its spread [1, 2]. One of the key interventions that received widened attention in the public health space was contact tracing [3]. Contact tracing is a process whereby individuals who are perceived to have come into contact with an infectious disease or an infected person are identified and subsequently isolated for close monitoring for a period of time, per the incubation period of the disease, in order to avert further transmission of the disease [4]. In the United States of America for instance, this practice is required for tuberculosis (TB), HIV, and other sexually transmitted infections [5]. Ghana was no exception in the implementation of contact tracing in tackling the COVID-19 threat [6].

Despite the global adoption of contact tracing in the control of infectious diseases [7, 8] including Ebola in Africa [9], the practice is bridled with numerous challenges. For instance, in Eastern Washington of the United States of America, people sent threats to contact tracers due to misinformation on social media because contact tracers were seen as spies [10]. Some contact tracers were also attacked in some communities in India [11]. In some African countries such as South Africa, Ethiopia, the Democratic Republic of Congo, and Mozambique, contact tracers encountered challenges such as inadequate human resources, workload, long turnaround time of COVID-19 test results and stigmatization [12, 13].

Despite the challenges associated with contact tracing [14], in developing countries such as Ghana, it remains a key public health intervention strategy in dealing with infectious diseases [15], as healthcare systems could capitulate during epidemics. While the developed world has advanced its contact tracing techniques by employing technology [16, 17], most African countries still rely on the traditional or manual contact tracing approach whereby (potential) contacts are physically traced by healthcare personnel in their communities. Hence, it is pertinent to identify the bottlenecks associated with contact tracing in the Bono region of Ghana to help improve contact tracing activities for general emergency management and response to issues of public health concern in the future.

In spite of the perceived challenges, a total of 27, 577 COVID-19 cases, representing 60.1% out of the 45, 857 total cases were reported in Ghana through contact tracing, as of mid-September, 2020 [18]. Thus, the absence of contact tracing could have led to a spike in COVID-19 cases in the country and possibly caused the loss of more lives. Moreover, although contact tracing has its challenges, it does offer useful insights into how experiences from the exercise could be leveraged for effective future pandemic control and prevention. In Germany, for instance, health authorities leveraged existing local infrastructure to get ahead of the COVID-19 pandemic [19]. Moreover, the need for innovative strategy in contact tracing through the deployment of technology has been highlighted as one of the opportunities that COVID-19 contact tracing has brought to bear [20].

The Bono region shares a border with the Ivory Coast, hence, it is susceptible to cross-border transmission of infectious diseases such as COVID-19. As a result, there is a need to strengthen contact tracing efforts in the region. Okertchiri [21] agreed that the first COVID-19 case recorded in the region was imported from the Ivory Coast, through the Jaman North district border, with prompt contact tracing playing a key role in halting the community spread of the disease. Hence, challenges that confront contact tracing in the region ought to be identified to inform policymakers on how best such challenges could be addressed in the future, not only for the region but the country at large. Also, opportunities that could be harnessed for improved future contact tracing activities needed to be ascertained.

Moreover, there is limited literature on COVID-19-related contact tracing, especially on its associated challenges. Various studies have looked into compliance with social distancing and other prevention protocols among the homeless, slum dwellers, the transport sector, health workers, and the general public at large [22, 23]. Other studies have also focused on the use of alternative medicine in the prevention and management of COVID-19, as well as the predictors of COVID-19-related stress [24, 25]. Although few studies have highlighted some of the challenges that COVID-19-related contact tracers faced in Ghana such as the irregular supply of personal protective equipment and remuneration, refusal of some contacts to test, delays in receiving test results, and poor coordination of the whole process [26, 27], opportunities for improving contact tracing have not been explored alongside these challenges in many studies.

Hence, this study explored the challenges encountered by contact tracers in the Bono region of Ghana as well as the opportunities that COVID-19-related contact tracing presents which could be leveraged for effective contact tracing in the region in the future.

## Materials and methods

### Study site description

The study was conducted in six selected districts that recorded the highest COVID-19 cases in the Bono region of Ghana. Located in the North-Western part of Ghana, the region comprises twelve (12) districts with Sunyani as its capital. It shares borders with the Savannah region to the North, the Ivory Coast to the west, the Bono East region, Ashanti to the East and Western North, and the Ahafo region to the South. The region consists of 11,481 km square of land with a total population projection of 1,082,520 [28]. The Bono region was selected for the study because it shares borders with the Ivory Coast, making it vulnerable to cross-border COVID-19 cases since the first case recorded in the region was from the Ivory Coast [21]. Hence, there is a need for a fortified strategy and preparation in contact tracing in the region for future pandemic control.

### Study design and period

An exploratory qualitative study design, with a focus group discussion (FGD) approach, was used to conduct the study, from September to October 2021. A focus group consisted of contact tracers on the field to trace, identify, isolate, and monitor individuals who came into contact with suspected COVID-19 persons. A minimum of six and a maximum of ten persons constituted a focus group. The study population involved all contact tracers in the six selected districts.

### Participants’ selection process

A total of 39 contact tracers, grouped into six focus groups, were interviewed. **To be included in the study, a contact tracer had to be at least eighteen (18) years of age and had worked as a contact tracer in any of the selected districts for at least twelve weeks**. However, any contact tracer that met the inclusion criteria but was either critically ill or had traveled out of the district at the time of the data collection was excluded. In each district, the list and contact details of contact tracers were obtained from the District Disease Control Officer. Potential participants were then contacted telephonically and screened for eligibility, availability, and participation.

### Data collection procedure

A semi-structured focused group moderation guide was used to collect the data. The guide consisted of questions on the socio-demographic characteristics of participants, the challenges they faced during contact tracing, and the opportunities that exist for the improvement of contact tracing. The questions were drafted in the English language and translated into Twi. The tool was pre-tested with six contact tracers, grouped into a single focus group, in a selected district in the Bono East Region that shares similar geo-political characteristics with the Bono Region.

In each district, data collection proceeded after the formation of focus groups. Participants were brought together at the premises of the various district health directorates (DHDs), for focus group formations and discussions. Each focus group was assigned an alphabetic code (e.g. GA, GB, GC, GD, GE, and GF; denoting districts A, B, C, D, E, and F respectively). Alphanumeric codes (e.g., A1) were then assigned to members of the groups for easy identification. The interviews were conducted in secluded rooms within the premises of the various health directorates to avoid distraction. Depending on the preference of the group, interviews were either conducted in English or Twi. Four out of the six FGDs were conducted in English and the other two, in Twi. Each interview session was audio-recorded and lasted about an hour.

### Data analysis

The inductive thematic analysis approach was employed to analyse the data. The approach ensured that the researchers did not have preconceived themes before the analysis [29]. The data that was collected in the Twi language was translated into English during the transcription process, by a qualified translator. All the transcripts were subsequently transcribed verbatim. The transcripts were reviewed by comparing them with the voice recordings. Afterward, all transcripts for each district were read to obtain some familiarity and examined for themes relevant to the research questions. The transcripts were then imported into ATLA S.ti version 9.0 for in-depth content analysis. Color coding of the text was done by two independent coders for theme formation and categorisation. To address discrepancies that emerged during the double coding process, the two coders i.e. the Principal Investigator, the team lead, and the independent coders met to review the codes. Codes with similarities in meanings were then merged while those with distinct meanings were treated as independent sub-themes. Sub-themes were developed using the most repeated words or phrases for each category of codes.

### Quality control processes

To elicit credible responses, rapport was created with the contact tracers prior to the discussions to ensure that they felt comfortable. Also, we reverted to some of the participants that were reachable within each focus group with the transcripts to confirm that our translations and transcriptions were not misrepresented. Two peer qualitative researchers were also tasked to review the transcripts to authenticate the translations before data were analysed. Furthermore, there was documentation and description of the methods in detail to ensure that the study could be replicated. We also avoided any potential bias that might influence the opinions of the participants by eschewing preconceived ideas during the discussions. Lastly, each participant was allowed to describe their challenges and opportunities independently without any interference from the researcher.

## Results

There were 39 contact tracers involved in the study, comprising 29 males and 10 females. The ages of the participants ranged from 27 to 50 years. All participants were public health officers with a tertiary level of education. **Twenty-six (26) of the discussants had worked for more than five years as public health officers within their respective districts and the rest had worked less than 5 years in their respective districts.** Out of the total number, 19 were married. Also, 24 of the 39 participants were Christians and the rest were Muslims.

The thematic results were grouped under two major themes; challenges associated with contact tracing and opportunities for improving contact tracing. The discussants reported twelve challenges that hindered effective contact tracing in the Bono region. These include inadequate personal protective equipment, harassment by contacts, politicisation of the discourse around the disease, stigmatization, delays in processing test results, poor remuneration and lack of insurance package for contact tracers, inadequate staffing, difficulty in locating contacts, poor quarantine practices, poor education on COVID-19, language barrier and transportation challenges. With reference to opportunities for effective future contact tracing, discussants identified four opportunities. These include cooperation, awareness creation, leveraging knowledge gained in contact tracing, and effective emergency preparedness for future pandemics.

### Challenges faced by contact tracers

#### I. Inadequate personal protective equipment (PPEs)

Inadequate PPEs was one of the major challenges reported by all the participants. All of them agreed that although they were provided with some PPEs, they were inadequate. Gloves and nose masks were the only PPEs supplied to contact tracers. Sometimes, these PPEs were insufficient to effectively carry out their duties on a daily basis. Thus, the contact tracers, on some occasions, would have to purchase personal gloves and nose masks for their safety. A participant remarked that:

*We have a challenge with PPEs such as the [nose] mask. They are not enough. So, we have to buy it ourselves. Although the situation has improved, they always prioritised those in the intensive care unit so we do not have access to those ones. And the gloves are a bit of a challenge, we do not have enough to use, so mostly we have to rely on our pocket money to buy some of the gloves in handling the samples. (GC, male, 29 years old)*

Another participant also made similar comments on the quality of PPEs available in his district. He said:

*Initially, PPEs were not enough, the only available ones were the nose mask and gloves but the one that covers the face wasn’t available and we had to go with it that way, which posed a risk to us [contact tracers] but now, the situation has improved a bit, although we still don’t have those that cover the entire face. (GB, male, 37 years old)*

#### II. Harassment of contact tracers

Harassment from contacts and their relatives was another challenge reported during the discussions. Fourteen of the discussants alluded to this challenge. Some contacts solicited food stuff from contact tracers since they requested them to quarantine. Others also demanded their test results from the contact tracers. Some contacts also verbally abused and threatened to sue the contact tracers. A participant in District A remarked that:

*The psychologist faces a lot of harassment, whenever she calls a contact to calm them down before contact tracers go to the house, they start calling her and demanding stuff from her to the extend she had to block some of the contacts. Some even demand the results of their test from her, because she contacted them first. Sometimes they ask her, how she got their contacts and threaten to sue her for getting their contact without their permission. These are some of the challenges she goes through. (GA, female, 39 years old)*

Another participant narrated receiving verbal abused from contacts during call conversations to counsel them before assigning contact tracers. A respondent in District C noted that:

*We traced one case and I tried to counsel him before he was quarantined, then he started saying to me, ‘that thing about COVID-19 that you have gotten is your own problem’. Sometimes, verbally. you are victimised. They insult you and cut the [telephone] line and that is when you are not physically present. Imagine if you were with the person at the time. How they listen and react on the phone tells you that they don’t accept it [COVID-19]. So, if you are a contact tracer, you are at risk, they will insult you and abuse you. (GC, male, 37 years old)*

#### III. Politicisation of the discourse around the COVID-19 outbreak

The politicisation of the discourse around the disease was another challenge encountered by almost all contact tracers, as it was passionately reported by each group. Some contacts requested the political affiliations of contact tracers before accepting to cooperate. This was because they believed that the government had an ulterior motive for how the disease was managed. A discussant in District E remarked that:

*Politicising the disease is a challenge to us [contact tracers]. This is because you will get to a contact’s home and they start to politicise the entire process [of contact tracing] and they begin to ask you which party you belong to. (GE, male, 43 years old)*

Another participant from District C had a similar experience to share. He said:

*I think politicians educating the public on the disease, in the beginning, was a big problem for us [contact tracers]. It made the people believe that the government had a hidden agenda, so some of them [contacts] are very rude to us because they think we are just helping the government to implement its agenda. If we allow politicians to educate the public first and the general public mistrust them, then you, the health worker, are the one to be in trouble because you are the one that will go to them. (DC, male, 37 years old)*

#### IV. Stigmatisation

Seventeen discussants, from all six districts, reported stigmatisation as one of the difficulties they encountered during contact tracing. It was revealed that both contact tracers and contacts experience it. It was realised that most contacts were unwilling to provide information about their potential contacts or accepted contact tracers because of possible stigma from society. One participant from District D said:

*I think stigmatisation is one of the major challenges and is still there. We have a psychologist who does the calling and informs you that you have contact with this person. The moment they here contact with this person, you will trace but will not get them. Sometimes just the fear alone to come out and even give you the details you need is a problem. Because he/she fears that, when he gives out the information and others get to know this person is a contact to the positive case, they will run away from them. There was a situation where everybody was running away from the contact because they thought he has brought the disease. So, stigmatisation is a big challenge. (GD, male, 35 years old)*

Another participant explained how possibly exposed individuals to the disease hid because of the fear of stigmatisation. She explained that:

*One major challenge we face, especially when the disease was discovered and spreading to other districts, is stigmatisation. People who had the disease were stigmatised. So, when you are going to trace a contact about a particular case, it is very difficult. People are not willing to own up to the responsibility to say I am a contact to this client. Because they feel if they disclose that, they will also be stigmatised, it is a challenge to us, the contact tracers in identifying who a real contact is. Even some health workers have been ejected from their homes due to stigmatisation. (GE, female, 37 years old)*

#### V. Delays in processing test results

Delays in receiving test results were another challenge that affected contact tracing. Twenty-two of the discussants complained that delays in processing test results as a result of inadequate testing centres made it challenging for them to continue the quarantine process. One of the discussants from district A explained that:

*The testing centre is a challenge to us as a district. We have sent about 27 samples, and it is almost one and a half months now the results are not in yet. One of them was sick, but we don’t know the status of that person till now. Although we have testing centres here, they are doing internal testing, so we have to send our samples to Sunyani [the capital of the Bono region] before they are sent to the central laboratory, which delays a lot. (GA, male, 33 years old)*

Another participant from District D reported similar complaints about the delays in processing the testing results. The situation, on some occasions, led to misunderstandings between the contacts and the contact tracers. He said:

*Sometimes, you can send samples for testing but the results will not come early. This makes the contacts start complaining, especially when they have no symptoms. They will tell you they don’t have any disease which is why you cannot tell them their results. (GD, female, 41 years old)*

#### VI. Poor remuneration and lack of insurance package for contact tracers

Poor remuneration and lack of insurance packages for contact tracers were other challenges highlighted by almost all the discussants. A discussant revealed that the government had promised a 50% allowance to all frontline health workers and an insurance package for those who had contracted the disease in their line of duty. However, some health workers were yet to receive them as at the time of the data collection. Most of the participants became emotional during a discussion on remuneration. A participant recounted that:

*The government promised to give a 50% allowance to all frontline health workers; as we speak, some of us have not benefited from it. We don’t know whether the government selected its contact tracers. I am sure our names were submitted but out of the names submitted, they chose what they wanted and paid them, and we were side-lined. Although I still do the work I have something in mind. I will do the work but will not dedicate all my time. (GB, male, 35 years old)*

A similar sentiment was shared during one of the discussions. The problem of poor remuneration had worsened to the extent that some contact tracers threatened to quit working. A discussant had this to report:

*People who were much involved in COVID-19 did not receive the 50% at all. We were here and people who were not involved rather had it. Whiles those who are more involved don’t get the 50% and is a form of demotivation. If Ghana is going to record more cases in which more contact tracing is going to be done, then they need to solve this issue. If not, they should be sure that nobody will volunteer to be a contact tracer. (GE, female, 37 years)*

#### VII. Inadequate staffing

According to some nineteen discussants, the increasing number of cases led to an increased workload on contact tracers since the staff strength remained the same. A discussant reported that:

*It is tedious and a bit challenging because cases are increasing, the number of contacts has to also increase, and you have to trace every day. So, I will say the workload is too much. And also, sometimes you will go to the house but won’t meet the contact, they have already left to the farm and you have to go there several times before you will meet them. (GF, male, 45 years old)*

Another participant also explained how workload increased at the peak of COVID-19. He narrated:

*It got to a time when the workload was very high, especially at the peak of the outbreak when we had more cases. This gave the extra duty to us [contact tracers] and sometimes you need to go beyond office hours to carry out contact tracing and it was a challenge. (GC, male, 36 years old)*

#### VIII. Difficulty in locating contacts

A total of twenty-four discussants, from all six districts, reported difficulties in locating contacts. They complained that some of the contacts gave false information and inaccurate contact information. A participant recounted that:

*One thing we have generally in our country is a poor address system, although you will be given the address of the contact, you are unable to trace it because the street name is not clear or correct. And you will find it difficult in locating that particular person. Sometimes you will be given a particular number to call, and you will try calling but due to a bad network, you will not reach the person. It makes it difficult in locating contact as well. (GD, male, 36 years old)*

Another participant expressed similar concern by saying:

*Locating a contact is a bit more difficult here than that in a rural area. Sometimes you can be giving the address to the house but because the house is not numbered you will find it difficult to locate it. You have to call the contact before they will direct you to the house but if you are not lucky and they don’t pick up your call, then it becomes very difficult.* (GA, *male, 34 years old*)

#### IX. Poor quarantine practices

Fifteen of the participants reported that the practice of home-based self-quarantine posed a great challenge to contact tracing. This is because some of the contacts lived alone and had to physically interact with the rest of the community members to access some basic services such as access to food and water. A discussant explained that:

*With regards to the quarantine, the fact that they have to stay in the house themselves brought about another challenge of mingling with other people in the society whether they like it or not. For instance, some stay alone and he has to go to the market or to a food joint for food. So, they end up exposing others to the disease and it is difficult to trace those people [they mingled with] at the market. Some also stayed in compound houses and used public toilet facilities. All these [practices] exposed others to the disease. The best way is to get centres to quarantine them. (GB, male, 32 years old)*

Another discussant from District F explained:

*Some of the contacts lived in houses that did not make the work [contact tracing] easy at all. Some lived in compound houses and still mingled with people in the house, although they would tell you [the contact tracer] that they have been indoors throughout. (GD, female, 34 years old).*

#### X. Language barrier

Although language barrier was not a major challenge in all the districts, it was highlighted by four discussants in two districts. Hence, some contact tracers, sometimes, had to get an interpreter or another contact tracer who was familiar with the language of the contact. A discussant explained that:

*One challenge was a language barrier, I remember one of my contacts did not understand English or Twi, which made it very difficult for me to communicate with her, so, we needed to find someone who understand the language to serve as an interpreter. (GE, female, 29 years old)*

Another continued:

*There are a lot of settlers in this district so sometimes you do not understand their language. That makes the work difficult as you need to find an interpreter. (GC, male, 34 years old)*

#### XI. Poor public education on COVID-19

Nearly half (18) of the participants highlighted that inadequate information about the disease made contact tracing difficult. It was revealed that most contacts lacked proper health education at the initial stage of the disease outbreak and this influenced their reception to contact tracing. One discussant narrated:

*During the first wave of COVID-19, contact tracing was difficult because most people did not get enough information with regard to the disease. People who were contacts with a positive case did not understand the reason why they had to be quarantined for you to come and pick samples. You get to a contact’s home and the person is already gone instead of staying in the house. He/she is already out into the public. Either he is gone to work, a funeral or any occasion. So, the first phase of COVID-19 contact tracing was very difficult.* (GC, f*emale 39 years old*)

Another participant explained that:

*I would say poor education about COVID-19 is a major challenge to us [the contact tracers]. You get to a house and you need to explain all over why you are there and the need for the contact to cooperate with you. This makes the work extra harder as people have limited knowledge of the disease. I think the health promotion and education unit [of the Ghana Health Service] needs to up its game. (GB, male, 33 years old)*

#### XII. Transportation challenges

Despite not being reported as a major challenge, issues related to transportation were reported by three participants from District B. One of them said:

*The means of transport is a challenge to us, as at when we receive test results from the testing centre, we are supposed to rush into the community. Sometimes there might be a vehicle but no fuel so you have to go by motorbike to the community, which is not supposed to be so. We are not supposed to use a motorbike to convene the sample to the testing site from the household. But the district is having only one vehicle which is sometimes busy. (GB, male, 40 years old)*

### Opportunities for effective future contact tracing

Participants in the study recommended and identified opportunities that must be leveraged to help improve future contact tracing. These include cooperation, awareness creation, leveraging on previous knowledge in contact tracing, and effective emergency preparedness for future pandemics.

#### I. Leveraging cooperation from community leaders

Twelve of the participants revealed that the communities, with the support of their leaders, were cooperative and receptive. Hence, strengthening partnerships in such communities could help ease some of the initial challenges that contact tracers face. A discussant explained:

*The opportunities were great. For the health sector, everybody was involved from the DHD to the sub-district level because more awareness creation was done. The community leadership was also involved and we were also fortunate to have a minister coming from here, so the people wanted to cooperate so that his name would not be put in the mud. If we can build on these structures going forward and not only wait for another pandemic before we go for their help, I think it will help us a lot. (GA, female, 39 years old)*

Another participant echoed:

*The community cooperated with us except for a few ones, but generally because of the education from the health promotion unit and the information department they got to understand that, contact tracing was important, so I am of the belief that once they now know us as public health officers, we should keep our presence on the ground and not wait till when there is another disease outbreak. (GD, male, 47 years old)*

#### II. Leveraging COVID-19 awareness creation

Sixteen participants believed that the creation of awareness on COVID-19 could help improve contact tracing in the future. Discussants agreed that communities have become more aware of the disease as a result of constant education about the disease. Hence, authorities ought to capitalise on the opportunity to create more awareness of pandemics and educate citizens on the role of contact tracing. This will help improve the work of contact tracers in the future. One explained:

*Because of the education from the health promotion unit and other units, people have understood what the thing [COVID-19] really is. I think we should use this opportunity to keep educating communities on pandemics and other disease outbreaks and how contact tracing is a key public health activity. We should not just dissolve these teams. That is my opinion though. (GF, male, 35 years old)*

Other participants acknowledge that, due to the outbreak, they had easier access to the radio and information centres to educate the general public. As such, such activities should be continued: One explained:

*Because of the COVID-19 pandemic, most information centres and radio stations were calling the health staff to educate the general public on its prevention. It makes the health promotion work become easier, because, at first you have to be pleading for air time but because the pandemic was high and people wanted to learn more, the radio stations were rather approaching the workers. I think if we can encourage these stations to make such programmes part of their schedules, contact tracing would be more understood by the public and there will be no challenges in the future. (GC, male, 32 years old)*

#### III. Leveraging knowledge gained in contact tracing

According to some eight discussants, the experience gained through COVID-19 contact tracing and previous knowledge in contact tracing constitutes a crucial factor for effective future contact tracing. The data collection revealed that most of the contact tracers with prior experience in tracing contacts with tuberculosis were able to effectively manage the challenges associated with the job. Thus, the involvement of those COVID-19 contact tracers during future outbreaks could help halt the spread of such diseases in time. It could also reduce the period needed for training contact tracers during emergencies. One of them explained:

*I think one opportunity we have is prior knowledge in contact tracing as a public health officer. Because we always do contact tracing for infectious diseases such as TB, measles, and other diseases, contact tracing is not new to us. So, in the future, they can still rely on us. (GD, female, 29 years old)*

Another participant also revealed that adopting effective measures from other countries helped with COVID-19 contact tracing. She pointed out that such experience could come in handy during future pandemics. She noted that:

*Before Ghana recorded it [COVID] cases, we received information from outside Ghana because they have started. So, we had enough capacity building before we recorded our first two cases. This awareness and capacity building have continued and helped quicken our knowledge which helps us go back to our previous contact tracing. Because the diseases are not the same, although you may have additional knowledge about how to do contact tracing for tuberculosis (TB) and oral polio vaccine (OPV) so more knowledge was given and can be applied to other diseases in the future. (GB, female, 36 years old)*

#### IV. Effective emergency preparedness on the part of health authorities

Almost all the groups noted that the preparation by Ghana Health Service (GHS) for the fight against COVID-19 was inadequate. This was revealed in the challenges encountered with the supply and distribution of PPEs and personnel. They believed that the GHS should learn from these experiences and prepare adequately for future pandemics. One discussant explained:

*When COVID-19 actually came, we [the Ghana Health Service] were not prepared, which is why we faced a lot of challenges with contact tracing in the beginning. The PPEs were not there, yet we had to work. So, if the authorities could learn their lessons, I think we will be better prepared for the future. (GE, male, 36 years old)*

Another participant retorted:

*If you look at the number of challenges we had, it is a clear sign of non-preparation in terms of leadership. In this country, we always wait until things get out of hand before we act. Although with COVID-19, we acted a bit fast, we were still not prepared. When the disease broke out in China in 2019, we should have been able to project when it will get into Ghana and begin our preparations towards it, but we were hoping that it won’t get here and it did. So, we should learn lessons from this and prepare well for the future. (GB, male, 37 years old)*

## Discussion

### Challenges associated with contact tracing

One of the challenges that contact tracers faced in the course of their duty was inadequate personal protective equipment (PPEs) such as face masks and gloves. This was experienced especially at the initial stage of the outbreak. Despite their usefulness for the effective conduct of contact tracing, the PPES were often in short supply. Contact tracers, therefore, had to purchase these essentials for their safety. The issue of inadequate PPEs was reported by most African countries such as South Africa, the Democratic Republic of Congo, and Mozambique [12, 26]. In Ghana, and in the Bono region especially, this challenge was associated with the high demand for PPEs in developed countries since the fight to control the pandemic was global, thus, the production of these items could not meet its export demands. Also, countries such as Ghana had not begun local production of some PPEs, hence, coupled with the closure of borders, the importation of PPEs could not materialise [30]. The shortage of PPEs, thus increased the risk of exposure to the virus for contact tracers since they were poorly protected. The situation created fear of contracting the disease in the minds of contact tracers, thereby hampering effective contact tracing [31]. This finding highlights the need for promoting and upscaling local production of basic medical essentials in the region and the country at large, such as PPEs. This could help to avoid shortages during future contact tracing activities.

Harassment from contacts was another challenge encountered by contact tracers in the Bono region. This involved physically and emotionally abusing contact tracers with threats and material demands by some contacts. This finding is consistent with the findings of Smith et al. [32] in North Carolina where some contact tracers were harassed by contacts with several calls and physical threats. Also, in Sierra Leone, Olu et al. [33] found that contacts sometimes drove contact tracers out of their homes or hide on seeing them during the Ebola outbreak. This could be attributed to what Stone [34] reported in Eastern Washington where misinformation on social media led to the belief that some contact tracers acted as spies, thus, were abused by some Native Americans. Such treatment meted out to contact tracers could discourage them from volunteering during future pandemics. Effective public education during disease outbreaks is, therefore, to garner public support and confidence in contact tracers within the region.

In addition, on the issue of politicisation of the disease, it was found that some individuals believed that contact tracing had an ulterior political motive. Hence, some contacts were hostile towards contact tracers. According to Bandura [35], people mostly model those they trust during behaviour change. Hence, as contacts were mostly politically divided along the two major political leanings of the country, thus the National Democratic Congress (NDC) and the New Patriotic Party (NPP), held different views about the pandemic and the presence of contact tracers. Also, the creation of a COVID-19 task force by the major opposition party, NDC, led to hostility towards the official government contact tracing team. Other political comments from the opposition parties further complicated the fight against the disease [36].

A similar situation was reported in the United States where the political battle between the president and the scientific community on whether to practice social distancing or not affected the fight against COVID-19 and contact tracing [37]. Furthermore, politicisation of the fight against COVID-19 such as the wearing of masks and practicing social distancing affected the fight against the disease in Ghana as also reported in the USA [38]. It could also be said that politicisation of contact tracing worsened as the outbreak coincided with the period of electing both the president and members of parliament for the country. Hence, the leading political parties politicised most COVID-19-related activities, including contact tracing, for political gains. It is therefore prudent for the region and the country at large to depoliticize emergency health issues such as the COVID-19 outbreak since it has the tendency to thwart the efforts of salient public health interventions such as contact tracing.

Furthermore, on the challenge posed by stigmatization in the Bono region, it was observed that both contacts and tracers were stigmatised in some communities. Some community members shunned the company of tracers and contacts because of negative pre-conceived ideas about the pandemic. This is similar to what was identified among the Lebanese population infected by COVID-19 individuals who experienced stigma from community members [39]. This was also reported during the Ebola outbreak where contact tracers were stigmatised because some individuals believed they had an increased risk of infection [40]. Stigmatisation caused some contacts and infected persons not to own up or mention the names of potential contacts. This hampered the contact tracing process. Therefore, there is a need for intensified public education during pandemic outbreaks to minimize stigmatisation to achieve effective contact tracing in the region in the future.

Moreover, the period for processing samples to arrive at the testing centres and informing the contact tracing team was overly extensive. This challenge has been reported in Ghana [26] and in South Africa [12]. Thus, there were delays in testing the samples, especially during the early stages of the outbreak. This was a result of a lack of specialised laboratory facilities with skilled staff. Hence, some samples had to be transported to some centralised laboratories and this delayed the process and feedback on test results to contacts [12]. The delay in receiving feedback on test results could have led to the denial of results by contacts, loss of interest for updates, or in some cases, suspected contacts unwilling to be tested [26]. The situation marginally improved when the number of testing centres was subsequently increased. Health authorities in the Bono region and Ghana in general, thus need to invest in modern laboratory infrastructure to be better prepared to handle future pandemics.

Poor remuneration and lack of insurance packages for contact tracers also hampered effective contact tracing in the Bono region. If contact tracers are better remunerated and insured, they will enhance their efforts in stopping the spread. Consistent with the findings of Tesfaye et al. [13], contact tracers in Ethiopia expressed concern about delays in receiving payments and this negatively affected their enthusiasm for the work. Contact tracing is laborious in nature, and having to do it during deadly epidemic outbreaks such as COVID-19 means that one has to be duly compensated. Moreover, as a deadly infectious disease, ensuring the lives of contact tracers to give off their best in such trying times is not out of place. The regional health authorities, therefore, need to offer better remuneration and insurance packages to contact tracers for effective contact tracing in the future.

Inadequate staffing was another challenge that confronted contact tracers. This increased the workload on contact tracers and thus, affected their work rate. As the number of COVID-19 cases increased, the number of contacts consequently increased without an increase in the number of contact tracing teams. This rendered the contact tracing work ineffective as some contacts could not be traced due to work overload. The situation hampered the fight against the disease. A similar incident occurred during the outbreak of Ebola in Africa where inadequate contact tracers created a high burden of contacts on the tracers [9]. Also, in Tanzania and the USA, inadequate health staff affected contact tracing during the initial stages of the COVID-19 outbreak, thereby increasing the workload on the contact tracers [12, 41]. For effective contract tracing in the future, the number of contacts assigned to a tracer by health authorities in the Bono region, and the country at large, need to be revised.

Difficulty in locating contacts was another challenge reported by discussants. Due to poor address systems, contact tracers found it difficult in locating contacts. In some instances, the addresses provided by contacts could not be traced anywhere whilst some telephone numbers could not be reached. Some contact tracers, therefore, had to ignore some contacts. This could have increased the spread of the disease. A similar situation was reported by Greiner and colleagues [42] during the Ebola outbreak in Liberia where issues such as a lack of a proper address system for contact persons, locations without street names, difficult terrain, and telecommunication unavailability made it difficult to locate some contacts. Hence, health officials in the region need to work hand-in-hand with other government agencies to ensure proper addressing systems in the region for easy identification and location of contacts during disease outbreaks. This is crucial because countries with good address systems locate cases quickly, enabling them to isolate patients and reduce the spread of the disease [43].

Poor quarantine practices exhibited by come contacts was another challenge confronted by contact tracers. The living conditions of some contacts made it difficult for them to be quarantined and this led to poor quarantine practices. Some contacts lived alone while others dwelled in compound houses, making it impossible to isolate themselves for quarantine and hampering contact tracing. Those who lived alone and needed some essential supplies were thus compelled to break the quarantine protocols and mingle with the general public.

Other contacts also lacked private toilet facilities in their homes and needed to make use of public toilets. Asiimwe et al. [26] had earlier reported similar findings in Ghana where contacts believed it was difficult to adhere to self-quarantine, especially getting food supplies and other necessities. Another study in Turkey reported that contacts believed they could not quarantine when people who believed had no symptoms of COVID-19 were allowed to gather for political rallies [43]. To forestall future occurrences, adequate designated quarantine centres and single-occupancy apartments need to be provided, if self-quarantine is to be enforced in the region.

Language barrier was another challenge that affected contact tracing. If contacts and tracers do not speak a common language, it makes communication very difficult. In the study area, some contact tracers reported this challenge as there were multi-ethnicities in some districts. In such instances, an interpreter was required to convey information to contacts. This could cause mistrust, and misinformation and hinder efficient contact tracing. A similar situation was identified in Wicomico County, Maryland, USA, where the language barrier made it difficult for contact tracers among non-English speakers [44] and in migrant communities [45]. Hence, in assembling a contact tracing team, the language proficiency of contact tracers should be considered by the regional and district health directorates before assigning them.

Lack of proper education leading to misinformation and misunderstanding on COVID-19 was also reported. Adequate information about the disease at the early stage helps people to understand the epidemiology of the disease and institute measures to prevent it. Inadequate information due to a lack of proper education on the disease was a major challenge at the initial stages of the COVID-19 outbreak in the Bono region. People relied on social media and non-experts for information that was, sometimes, inaccurate. In Turkey, inadequate information about COVID-19 pushed some contacts to demand testing but rejected treatment while other contacts only agreed to be tested if they would be allowed to live their normal lives without any quarantine [43]. It can thus be noted that inadequate information can create misinformation, stigma, and misconception among people. As reported by Graham-Rowe [46], the lack of adequate information during the Ebola outbreak in Liberia influenced some people to believe that contact tracers were trying to make up stories about the contacts or their relatives about having the disease. Hence, early expert-led education during disease outbreaks should be embarked upon by the regional health authorities to dispel negative reportage by non-experts in the media.

Lastly, the lack of means of transporting samples was another pertinent challenge mentioned by the discussants. In Ghana, although it was reported that drones were used in transporting samples to the testing site [47], some districts in the Bono region still had challenges transporting samples. A similar challenge was reported in Sierra Leone during the Ebola outbreak where specimen managers had to use their personal vehicles to transport Ebola specimens to laboratories [48]. Also, in Uganda, Ebola samples arrived late in laboratories for testing due to transportation challenges [49]. Hence, increasing the number of testing centres by setting up laboratories in every district of the region and provision of adequate vehicles dedicated to contact tracing during pandemic control could forestall this challenge in the future.

### Opportunities for improved future contact tracing

In this study, the high level of cooperation discussants received from community leaders and the general public at large, albeit with few hesitations was seen as one of the avenues that could be explored to improve contact tracing in the future. Cooperation makes contact tracing easier and motivates tracers in executing their duty. It was reported that harnessing the cooperation received from community leaders in times of disease outbreaks could ease community entry and acceptance for effective contact tracing. This finding was consistent with what Nkansah [50] reported in Ghana and McCarten-Gibbs [51] in the United States where a high level of cooperation from community members and faith-based organisations led to effective contact tracing. The cooperation could be a result of the trust in the health sector over the years owing to frequent community outreach programmes [52]. It could also stem from the fear surrounding the disease at the onset or as a result of the high awareness that was created about the disease in the Region. Effective liaison with community leaders and members during pandemics could thus open them up for improved contact tracing in the region.

Another opportunity that contacts tracers agreed could be leveraged to improve future contact tracing efforts is high community awareness of COVID-19 contact tracing. They explained that the free air slots that were provided by radio stations and information centres in most communities in the Region for COVID-19 sensitisation helped to create much awareness of the disease and the need for contact tracing. Hence, communities have become accustomed to the practice of contact tracing. However, such an important public health education needs to be periodically embarked on in communities to sustain awareness. This assertion has been corroborated by Smith and Aerts [53] where education and awareness about COVID1-9 within families and communities provided opportunities for effective contact tracing [53]. This high level of awareness created on contact tracing could increase the level of cooperation needed from the general public against future outbreaks in the region.

Another opportunity reported is knowledge and experience gained through COVID-19 contact tracing. Possessing knowledge on contact tracing provides tracers with enough experience in their work. This implies that they can mitigate some possible challenges on the field. Similarly, Li et al. [54] reported that previous knowledge of contact tracing for diseases could help with future contact tracing activities. Moreover, experiences drawn from the Ebola outbreak where some contact tracers were involved helped them acquire the needed experience in COVID-19 contact tracing [55]. Hence, drawing on the experience of seasoned contact tracers instead of recruiting new teams during disease outbreaks could go a long way to improve contact tracing efforts in the region.

Lastly, the experience acquired from the challenges that confronted contact tracers during COVID-19 in the Bono region could help improve emergency preparedness for future pandemics in the region. For instance, sustained production and stocking of local PPEs such as face masks, hand sanitisers, and gloves could be encouraged to avert future shortages. In some African countries, for instance, governments leveraged existing infrastructure such as the polio geographic information system platforms for COVID-19 contact tracing and surveillance challenges [56]. Although contact tracers in the Bono region and the country as a whole leveraged existing health infrastructure for COVID-19 contact tracing such as the surveillance outbreak response management and analysis system and the community-based surveillance and geographic information [57], the experiences, and opportunities it brought, such as the use of drones for the transportation of samples and medical supplies [47], could be leveraged to improve future contact tracing activities.

Despite the efforts to ensure that our findings are credible, the qualitative nature of the study only allowed a few participants in a single region of the country to be interviewed. Hence, our findings should be interpreted with caution, as generalising them on the entire country might be out of context and misleading.

## Conclusion

We identified challenges associated with COVID-19-related contact tracing in the Bono region of Ghana while unearthing opportunities that exist for improved future contact tracing from the perspective of contact tracers. Hence, health authorities addressing these challenges and harnessing these identified opportunities could help improve contact tracing in the region in the future.

## Electronic supplementary material

Below is the link to the electronic supplementary material.

Supplementary Material 1

## Data Availability

All data generated or analysed during this study are included in this published article.

## List of abbreviations

COVID-19: Coronavirus Disease 2019
DHDs: District Health Directorates
FGDs: Focus Group Discussions
NDC: National Democratic Congress
NPP: New Patriotic Party
PPEs: Personal Protective Equipment
UHAS-REC: University of Health and Allied Sciences Research Ethics Committee
USA: United States of America.
