# Supplementary material for: Challenges and opportunities for improved contact tracing in Ghana: experiences from Coronavirus disease-2019-related contact tracing in the Bono region
Source: BMC Infect Dis. 2023 May 18;23:335. doi: 10.1186/s12879-023-08317-6 (PMC10193342; doi:10.1186/s12879-023-08317-6)
Supplement: Supplementary file 1 — Supplementary Material 1 [file 12879_2023_8317_MOESM1_ESM.pdf]

FOCUS GROUP DISCUSSION GUIDE

Title: Threats, Challenges, and Opportunities Associated with COVID-19- Related Contact Tracing in Ghana: The Case of The Bono Region

Background:

My name is Isaac Tachie Asare, a level 400 Health Promotion Student at the Fred N. Binka School of Public Health of the University of Health and Allied Sciences, Hohoe. I am conducting research titled "**Threats, challenges, and opportunities associated with COVID-19-related contact tracing in Ghana: The case of the Bono region"** in partial fulfillment of the requirements for the award of a Bachelor of Public Health degree in Health Promotion. This group discussion will last for about an hour and will be audio-recorded, with your permission. Kindly read the attached leaflet for your rights, benefits, and discomfort associated with this study and decide whether you would like to participate or not. Should you decide to participate, you will be required to append your signature or thumbprint on the same form. However, in case you do not wish to participate, you are free to walk away. Thank you.

Instructions:

i. You are to take turns responding to the questions. This will be done in no particular order. For you to be called, kindly raise your hand when a question is posed.
ii. You are to calmly wait for the one speaking to finish his/her submission before you also speak.
iii. You can add to what the person said or clarify it if you feel some details have been left out, based on your experience on the field, or raise an entirely new point.
iv. There are no right or wrong answers as you are all sharing your experiences on the field, which might differ from one person to another.
v. Before you respond to a question, kindly mention your unique ID so that we can relate what you say to you as an individual during the data analysis process.
vi. Before we proceed, kindly complete the background information section on the form given to you and hand it over to the assistant.
vii. The unique ID on your form (which has been written on a card for you) is what you will quote when you want to respond to a question.

PARTICIPANT ID……………….

Date of interview: ____ /____/____ (DD /MM/ YYYY)

BACKGROUND INFORMATION OF PARTICIPANTS

1. District: …………………………….
2. How long have you been working in the district………………..
3. Age of participants………………….
4. Religion
5. Marital Status
6. Sex
7. Ethnicity

Threats encountered during contact tracing

Q1. What were some of the threats you encountered during the contact tracing in your district? (Probe for risk of infection, stigmatization, mental health challenges, limited public compliance, and limited testing capacity)

Q2. Are there other threats you encountered that we have not mentioned in our discussion yet?

Challenges encountered during contact tracing

Q3. Can you share with us the challenges you encountered during contact tracing in your district? (Probe for lack of resources including limited human resources, communication barriers, transportation challenges, inadequate surveillance systems, resistance to quarantine, and poor motivation)

Q4. Tell me about any other challenge you faced that we have not mentioned in our discussion so far

Opportunities for improved contact tracing

Q5 From your experience, what do you think are some of the opportunities that could be leveraged to improve future contact-tracing activities in your district? (Probe for opportunities from the following sources: National, Regional, District, Communities, and NGOs). Also, probe for opportunities in strengthening the health system, increased awareness, technology adoption, and collaboration as opportunities that have been presented by the onset of COVID-19.

Q6. Is there any additional information that you would like us to discuss?

Thank you for your time
